# Factors influencing self-management of adrenal crisis in patients with adrenal insufficiency: a qualitative study

**DOI:** 10.1530/EC-24-0651

**Published:** 2025-04-28

**Authors:** Aldons Chua, Heather Yoeli, David Till, Umesh Dashora, Patrick Oyibo, William M Drake, Martin Cartwright, Sofia Llahana

**Affiliations:** ^1^ School of Health and Medical Sciences, City St George’s, University of London, London, United Kingdom; ^2^ Endocrinology Department, St. Bartholomew’s Hospital, Barts Health NHS Trust, London, United Kingdom; ^3^ Northern Lights Research Associates; Newcastle upon Tyne, United Kingdom; ^4^ Diabetes and Endocrinology, East Sussex Healthcare NHS Trust, East Sussex, United Kingdom

**Keywords:** adrenal insufficiency, adrenal crisis, self-management, hydrocortisone injection, parenteral glucocorticoid therapy, sick day rules, stress dosing

## Abstract

**Objective:**

Adrenal crisis is a life-threatening complication that requires urgent administration of parenteral hydrocortisone. Current patient education interventions remain ineffective, contributing to preventable hospitalisations and deaths. This study explored the lived experiences of individuals with adrenal insufficiency, focussing on the factors influencing self-management during adrenal crises.

**Methods:**

This qualitative study employed online semi-structured interviews with adults with adrenal insufficiency who had experienced at least one adrenal crisis in the past 3 years. Participants were recruited via patient advocacy groups in the United Kingdom. Data were analysed using an inductive thematic approach, allowing themes to emerge directly from the data without imposing predetermined categories.

**Results:**

Twelve themes, grouped into four overarching domains, captured individual experiences of managing adrenal crises: i) knowledge and experience; ii) tools and training; iii) psychological and emotional impact; and iv) support and dependence on others. Participants reported challenges including delayed diagnosis, difficulties recognising adrenal crisis symptoms and the complexity of the hydrocortisone injection process. However, prior experiences of adrenal crises, patient education and advocacy resources fostered greater confidence in self-management. Participants highlighted the need for simplified injection devices, clearer stress dosing guidelines, improved training for healthcare professionals and increased public awareness.

**Conclusion:**

Findings from this qualitative study emphasise that effective adrenal crisis management requires patient-centred, evidence-based interventions focussing on education, healthcare professional training and public awareness. Simplified hydrocortisone delivery devices and systemic reforms are crucial to supporting self-management and minimising preventable hospitalisations and fatalities caused by adrenal crises.

**Plain language summary:**

People with adrenal insufficiency face life-threatening emergencies called adrenal crises, which need urgent treatment with hydrocortisone injections. In this study, 12 people shared their struggles, including complex injection procedures, severe symptoms which made self-injection challenging and limited support to manage these crises effectively. Simpler injection devices, better information and improved training for healthcare staff could help people self-manage their condition better, prevent avoidable hospital admissions and save lives.

## Introduction

Adrenal crisis is a life-threatening emergency and the most severe manifestation of adrenal insufficiency, triggered when cortisol demand is not matched by adequate glucocorticoid and/or mineralocorticoid provision. Most patients with adrenal insufficiency require lifelong glucocorticoid replacement, typically hydrocortisone tablets taken 2–3 times daily. During illness or stress, patients need to follow ‘sick day’ or ‘stress dosing’ rules by increasing their glucocorticoid intake, and parenteral hydrocortisone is required during an adrenal crisis to prevent hospitalisation and death (1, 2). There is no universally agreed-upon definition for adrenal crisis, although most accepted definitions emphasise acute deterioration and profound impairment of general health, with at least two of the following signs and symptoms: arterial hypotension with systolic blood pressure <100 mmHg or relative hypotension (≥20 mmHg lower than usual), nausea or vomiting, severe weakness or fatigue, fever, somnolence, confusion or impaired consciousness, hyponatremia (≤132 mmol/L), hyperkalaemia and/or hypoglycaemia (more common in children), necessitating immediate parenteral glucocorticoids (1, 3, 4, 5, 6, 7) resulting in a marked improvement in symptoms within 2 hours post-parenteral glucocorticoid administration (1).

Approximately 6–8% of patients with adrenal insufficiency experience a life-threatening adrenal crisis every year (8). Prospective studies from Europe reported an incidence of eight adrenal crises per 100 patient-years (9, 10), while a recent retrospective US study found an incidence of 24 adrenal crises per 100 patient-years; 44% of patients had at least one adrenal crisis since diagnosis (11). Patients with primary adrenal insufficiency are at higher risk of adrenal crises compared to those with secondary adrenal insufficiency, with incidence rates of 5.2–7.6 and 3.2–5.8 per 100 patient-years, respectively (8, 12, 13). However, patients with tertiary adrenal insufficiency face the highest risk, with an incidence of 15.1 adrenal crises per 100 patient-years (13). The mortality associated with adrenal crisis is 0.5 per 100 patient-years due to rapid health decline, despite the preventable nature of such episodes (3, 9). Data on hospital admissions in the United Kingdom indicate that deaths related to adrenal crises are ten times higher than those caused by diabetic ketoacidosis (14).

The standard treatment for adrenal crisis requires immediate parenteral hydrocortisone administration, a glucocorticoid equivalent to cortisol, to restore physiological function before prolonged hypotension causes irreversible damage (1). In adults, the recommended initial dose is 100 mg of hydrocortisone given intramuscularly or intravenously, followed by 200 mg over 24 h via continuous infusion or 50 mg bolus every 6 h, in the hospital setting (1, 7, 15). Patients with adrenal insufficiency are required to be competent in reconstituting, drawing up and administering an intramuscular injection to self-manage an adrenal crisis; this process involves more than 15 steps and requires adequate vision and dexterity. The profound physical and neurocognitive impairment associated with an adrenal crisis, compounded by the complexity of the hydrocortisone injection, often hinder a patient’s ability to self-inject, even if fully trained, necessitating assistance from a caregiver or a healthcare professional (1, 3, 16, 17, 18, 19). An anxious caregiver may also find it difficult to perform this complex task, compounding the risk of mortality for the patient experiencing an adrenal crisis (19, 20).

Studies show that although over 70% of patients or their caregivers are trained and are in possession of an emergency injection kit (16, 21, 22, 23), fewer than 25% manage to self-inject or have this administered by a parent or caregiver (19, 21, 23, 24, 25), with rates as low as 12% in some cases (10, 24, 26). Allolio highlighted that up to 50% of adrenal crises-related hospitalisations may be prevented with effective self-management and timely self-injection (3). A study by Burger-Stritt *et al.* found that 38% of patients who self-injected at home required hospitalisation versus 73% who had to wait for a healthcare professional to administer their hydrocortisone (*P* = 0.008). However, a recent study reported that 41% of patients were unable to self-inject despite attempting to do so during an adrenal crisis (18). Evidence from our group further demonstrates that adrenal crisis management is complex and influenced by multiple factors, often beyond the patient’s control, including injection device-related barriers, external constraints and emotional responses such as anxiety and lack of confidence (19). The complexity of the multi-step hydrocortisone injection process has been cited as a major barrier by more than 80% of patients and caregivers (19, 27).

Despite various initiatives to enhance disease awareness and patient education, self-management of adrenal crises remains a significant challenge (5, 6, 17, 27, 28, 29, 30). Sixty percent of patients with adrenal insufficiency report reduced self-confidence in effectively managing a crisis, even after receiving adequate information and injection training (5). Current evidence provides limited guidance on effective self-management strategies to support patients during adrenal crises, highlighting a critical gap in existing practices and the need for approaches that empower patients to independently recognise and manage these life-threatening situations.

This study aimed to provide an in-depth understanding of patients’ experiences and perceptions of managing adrenal crises, with a particular focus on the factors influencing the administration of parenteral hydrocortisone in home or in community settings.

## Participants and methods

### Study design and research paradigm

A constructivist paradigm (31) guided the exploration and interpretation of patients’ diverse experiences and perceptions of managing adrenal crises, providing insights into how individuals understand, adapt to and navigate the challenges associated with managing these life-threatening episodes. We adopted the ‘Consolidated Criteria for Reporting Qualitative Research (COREQ)’ guidelines to design and report this qualitative study (32).

### Setting and participants

Patients with adrenal insufficiency were recruited via two UK-based Patient Advocacy Groups (PAGs): the Addison’s Disease Self-Help Group (ADSHG) and the Pituitary Foundation, with a combined approximate membership of 3,500 individuals. Using a convenience sampling approach, potential participants confirmed their eligibility and provided informed consent via a secure University Qualtrics® account to participate in the study.

#### Inclusion criteria

- Adults older than 18 years of age diagnosed with adrenal insufficiency on glucocorticoid replacement therapy, residing and receiving medical care in the UK.
- Experienced at least one adrenal crisis in the past 3 years requiring parenteral hydrocortisone, either self-administered or given by a family member, caregiver or a healthcare professional.

Given the lack of a universally accepted definition of adrenal crisis, we adopted a pragmatic approach, allowing participants to describe their experiences based on their own understanding rather than predefined clinical criteria. The occurrence of an adrenal crisis was determined based on the symptoms described during the interviews and assessed by the first author conducting the interviews.

### Data collection

Semi-structured interviews, each lasting up to 60 min, were conducted by the first author using a secure Microsoft Teams University account, between June and July 2023. An interview guide (Supplementary Material (see section on Supplementary materials given at the end of the article)) was developed in consultation with all authors and a patient advisory group based on the study objectives and was pilot tested with the first participant. Patients were invited to have a family member or caregiver join the interview, if they felt support was needed to help recall the adrenal crisis events, provided that the person was present during the crisis and both were comfortable discussing the experience.

### Data analysis

Analysis was conducted in parallel with data collection and video recordings were transcribed verbatim by the first author. Qualitative thematic analysis using the QSR NVivo 12 enabled a rigorous, inductive process of data familiarisation, coding and theme identification to capture shared underlying experiences among participants (33). To minimise interpretive bias, transcripts from five interviews were blind coded by HY, a qualitative researcher with no domain expertise in adrenal insufficiency, cross-checked with AC and validated by SL. Reliability was assessed by theme concordance and in discussion with the research team.

### Ethical considerations

The study was approved by the Ethics Committee at the School of Health and Psychological Sciences Health, City St George’s, University of London (ETH2223-1304). Each participant was assigned a unique study ID, and data handling complied with the Data Protection Act 2018, the General Data Protection Regulations 2016 and the Data Protection Bill, ensuring confidentiality and data integrity.

## Results

Twelve participants, all white European females residing in various locations across the UK, were interviewed for this study, including one accompanied by their next of kin. The mean age of participants was 43.8 years (SD = 10.2; range: 27–65), with a mean duration of adrenal insufficiency since diagnosis of 7.5 years (SD = 5.7; range: 1.5–17). Three participants lived alone, while nine lived with family members or partners. Nine participants had secondary adrenal insufficiency (four with acromegaly, three with Cushing’s disease and two with Sheehan’s syndrome), and three had tertiary glucocorticoid-induced adrenal insufficiency. Four participants were taking prednisolone, and eight were taking hydrocortisone for glucocorticoid replacement. All participants had at least one comorbidity and were on concomitant medication, primarily pituitary replacement therapies. None of the participants reported any visual, physical or motor impairment that could have affected their ability to self-administer hydrocortisone. On average, participants experienced 4.4 adrenal crises in the past 3 years (SD = 2.5; range: 2–9).

**Figure 1:**
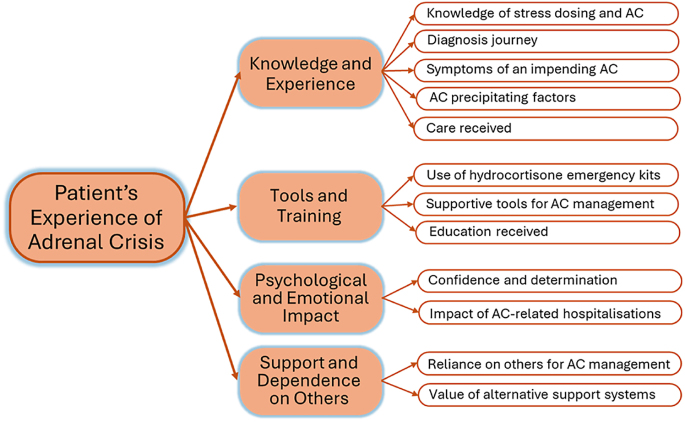
Domains and themes describing patient’s experience of adrenal crisis (AC).

Twelve themes captured patients’ experiences and perceptions of managing adrenal crises with parenteral hydrocortisone. These themes were mapped against four overarching domains: i) knowledge and experience; ii) tools and training; iii) psychological and emotional impact; and iv) support and dependence on others (Fig. 1). Participants reported varied experiences throughout their adrenal crisis journey, from the period before diagnosis to their most recent crisis. All participants had experienced at least two adrenal crises within the past 3 years, and five participants reported having five or more episodes during that period. Adrenal crisis-related symptoms varied among participants: two reported low blood pressure, six described loss of consciousness and one reported hypoglycaemia. Nine out of 12 participants stated that they attended accident and emergency during most, if not all, of their adrenal crisis events.

### Knowledge and experience

#### Knowledge of stress dosing and adrenal crisis

Participants had mixed views on understanding and defining adrenal insufficiency and adrenal crisis. Most participants reported that better understanding and awareness developed gradually over time as they learnt how to manage their condition and listen to their body:

*“I think I’m new to it, so I don’t know. I’m winging it because like I said, I’ve got so many conditions.”* (P9)

Some participants highlighted confusion around the term ‘sick day rules’ or ‘stress dosing’, interpreting it as applying only to actual illness, which led to reluctancy adjusting their glucocorticoids during situations such as mental exhaustion or physical injury, suggesting that:

*“I think they [guidelines] need to move away from the idea of sick dosing, and they need to move more of an idea of stress dosing … people would understand that there’s a need to kind of increase steroid coverage for more things.”* (P7)

#### Diagnosis journey

Some participants, especially those with secondary adrenal insufficiency, reported experiencing unrecognised non-specific symptoms of adrenal insufficiency for many years before receiving a formal diagnosis. The delay in obtaining an accurate diagnosis made it challenging for them to come to terms with their condition. One participant even perceived some of these symptoms as adrenal crises:

*“I was diagnosed with acromegaly 15 years ago, but I had symptoms for at least 3 years before that, probably more like five adrenal crises looking back* *… an adrenal crisis would’ve been coming on, and I hadn’t really realized.”* (P1)

#### Symptoms of an impending adrenal crisis

Some participants experienced obvious symptoms, with vomiting, nausea, lethargy and unresponsiveness being the most reported:

*“… my head drops. I can’t hold my body up. And then suddenly, my arms have gone over, and I don’t make sense. I’m gibberish and that’s the start.”* (P8)

However, others found certain adrenal crisis symptoms challenging to recognise, leading to feelings of hesitation and uncertainty about when and how to stress dose or self-inject hydrocortisone. This feeling was also shared by the next of kin to one of the participants:

*“It’s not black and white … it’s not binary. Is she in an adrenal crisis or not? I still don’t know.”* (P2-NOK)

#### Adrenal crisis precipitating factors

The most commonly reported precipitating factors that participants identified as triggers for adrenal crises were gastrointestinal infections (‘sickness bugs’) and other infections, cited by nearly all participants. Several also identified upper respiratory tract infections, such as common colds. Additional precipitating factors included non-adherence to glucocorticoid treatment, either by missing doses or failing to increase the dose during periods of illness and ‘sick days’, along with insufficient sleep, emotional distress, physical exhaustion and medication changes, such as switching from hydrocortisone to prednisolone. Notably, more than half of the participants reported difficulty recognising the warning signs or triggers of an impending adrenal crisis, leaving them unprepared to take preventative action, as this patient noted:

*“I’m always unsure what’s triggering, unless I’ve got other key symptoms that I know.”* (P9)

Learning from previous adrenal crisis was essential for some participants as it enabled them to better understand the precipitating factors of an impending adrenal crisis and manage subsequent crises more effectively:

*“I knew that I could increase my steroid.”* (P1)

#### Care received

Participants’ experience of the care they received from non-endocrine healthcare professionals was influenced by their perception of these providers’ knowledge of adrenal crisis and willingness to accept guidance, such as the emergency steroid card. While some patients praised the excellent care received from their general practitioners and paramedics, there was an overall sense of frustration with hospital and emergency care personnel:

*“I turn up to A&E and say, I’ve had my injection. They just go, oh, well, okay, whatever. Like a lot of the time, it’s not recognised as an actual serious problem.”* (P5)

### Tools and training

#### Use of hydrocortisone emergency kits

One participant found the hydrocortisone injection simple and straightforward, noting that her healthcare background likely boosted her confidence. However, most participants found the injection kit complicated and challenging to prepare, with a primary concern being the multiple steps required to mix, withdraw and inject hydrocortisone, especially in comparison to other devices such as adrenaline autoinjectors or insulin pens. Some participants expressed preference for the premixed hydrocortisone ampule, while other suggested that preparing and administering the hydrocortisone injection before symptoms worsened facilitated their success in self-managing adrenal crises:

*“I was kneeling on the bathroom floor, and I managed to mix the two together and then inject through clothing, because I knew I got to get it in really fast.”* (P6)

#### Supportive tools for adrenal crisis management

Participants reported that the tools that alert others to the risk of adrenal crisis, such as the emergency steroid card, printed guidelines, medic alert bracelet and red flag system, were helpful in managing adrenal crises. However, despite these alerts, they often experienced delays in the timely administration of parenteral hydrocortisone:

*“I have got this steroid card … some staff looked at it like, I don’t need that, I’m a medical professional, I know my stuff. But I’ve had some people [healthcare staff] who’ve been very appreciative of it …”* (P2)

#### Education received

Overall, participants found hospital training on ‘sick day rules’ and how to administer hydrocortisone injections, although not all received in-person training, and some found these sessions confusing. Online videos from patient advocacy groups were especially valued for reinforcing in-person sessions:

*“The endocrine nurses … were very good about explaining the sick day rules and what I need to do [in an adrenal crisis].”* (P1)

*“The explanation regarding like the sick day rules, that really confused me. The videos were more helpful …”* (P9)

Participants also appreciated the use of multiple teaching methods, face-to-face sessions, online videos and printed leaflets, which they found beneficial, as this patient noted:

*“… when you have memory problems, it’s impossible. So having something written as well as having the video, that’s been the best thing.”* (P3)

### Psychological and emotional impact

#### Confidence and determination

Participants reported that their confidence and understanding in managing adrenal crises had significantly improved overtime through reflection on past experiences, fostering greater determination and self-efficacy in preventing future adrenal crises whenever possible, as this participant highlighted:

*“So, I know in the future, not to take any chances [during an adrenal crisis].”* (P11)

#### Impact of adrenal crisis-related hospitalisations

Past hospitalisation experiences related to adrenal crises influenced participants’ perspectives and preferences for managing future episodes. Seven participants indicated that, if given a choice, they would avoid attending accident and emergency department during subsequent adrenal crises. While COVID-19 exacerbated fears and anxiety about hospital admission, some participants also expressed general discomfort with the hospital environment:

*“I’ve developed a fear of going to the hospital because since the pandemic, they won’t let anyone stay with you. And if I’m feeling that bad, I can’t advocate for myself.”* (P4)

### Support and dependence on others

#### Reliance on others for management of adrenal crisis

Overall, participants found it challenging to self-administer hydrocortisone injections during an adrenal crisis. Those living with partners or family members reported they had to rely on them to administer the injection, while participants who lived alone said they wait for paramedics or attend accident and emergency to receive hydrocortisone:

*“… my husband had to give me my emergency injection, and that was the first time he’s had to do that. In previous occasions, the paramedics have given me it.”* (P2)

#### Value of alternative support systems

Participants found various support systems valuable for managing adrenal crises, including social media, YouTube videos and resources from patient advocacy groups, with one participant noting that:

*“I have honestly learned more on there [facebook group] than I’ve learned from any medical professional.”* (P7)

Participants also highlighted the need for other support systems, especially for those living alone, which would help in managing adrenal crises:

*“I’ve got a dog, and she can sense when my cortisol level drops. She’ll try and get me to sit down, take extra tablets and she won’t let me move until I get better again.”* (P12)

## Discussion

This study explored the multifaceted experiences of individuals managing adrenal crises, highlighting the diverse clinical, interpersonal and psychosocial factors that influence self-management strategies for adrenal insufficiency. Findings suggest that a variety of factors, such as knowledge and experience, emotional resilience, available tools, the complexity of the hydrocortisone injection and support systems including caregivers and the healthcare system, significantly shape patient perceptions and actions during adrenal crises. These insights affirm the need for improvements in both patient education and healthcare support to enhance self-management outcomes.

### Knowledge and experience

Participants’ understanding of adrenal insufficiency and crisis varied widely, often shaped by individual journeys through diagnosis and self-management. These findings align with previous research, which reported a strong correlation between patients’ educational background and level of their understanding of self-management (34). Some participants, particularly those with acromegaly and Cushing’s syndrome, reported that their delayed diagnosis affected their understanding of the need for glucocorticoid replacement therapy and preventive strategies for adrenal crisis. Our findings support the importance of lived experience in building knowledge and self-management skills, supported by earlier research, describing this experience as a ‘*transitional journey*’ of learning and psychological adaptation through a process of ‘reeling’, ‘dealing’ and ‘healing’ (35).

All participants in this study had experienced at least one adrenal crisis episode. Precipitating factors and symptoms varied, with gastrointestinal infections, vomiting and diarrhoea being the most commonly reported, aligning with earlier research reporting this in over half of individuals experiencing an adrenal crisis (8, 18, 19, 24, 25, 26, 36). Some participants reported non-specific triggers, and others misinterpreted the terms ‘sick day rules’ thinking this applied only to physical illness, which led to uncertainty about glucocorticoid dose adjustments during situations such as psychological stress or other non-illness triggers. The lack of a clear definition of adrenal crisis may have contributed to participants’ difficulty in recognising the onset of a crisis, highlighting the need for a standardised definition and improved patient education to enhance symptom recognition and ensure appropriate management (1, 5, 19, 30).

Several participants in our study suggested changing ‘sick day rules’ to ‘stress dosing’ as this would improve patients’ understanding of precipitating factors. Indeed, recent publications are now using ‘stress dosing’ as the terminology to describe glucocorticoid adjustment for increased stress situations (10). Emotional stress is recognised as a significant precipitating factor to adrenal crisis, with 23% of 541 adult patients with primary adrenal insufficiency in a US study reporting this as a trigger (36). Recent guidelines from the UK recommend stress dosing with a double or triple dose of oral hydrocortisone for 1–2 days for psychological stress (15).

### Tools and training

Most participants reported difficulties with the hydrocortisone injection due to the complexity of the device, a challenge exacerbated by the physical and cognitive impairment during an adrenal crisis, leading to inability to self-administer the hydrocortisone injection. In a recent study involving 688 patients with adrenal insufficiency and caregivers, over 80% identified the complexity of the injection process as the primary barrier to effective self-management (19), consistent with findings by Burger-Stritt *et al.* (27). In addition, 38% reported difficulties accessing the correct hydrocortisone formulation or necessary equipment, such as needles and syringes, which in some cases prevented timely injection due to missing items or delays in obtaining prescriptions (19). Previous studies also reported low success rates of self- or caregiver-administered injections ranging between 12 and 24% among patients with adrenal insufficiency (10, 19, 21, 24), and 41% in a more recent study (18), despite the fact that most patients possess an emergency hydrocortisone kit and have been trained to use it (19, 22, 27, 37, 38).

Our study found that, while face-to-face patient education was helpful for most participants, many still felt confused and did not retain a lot of information post-session, especially those with memory impairment. Participants valued supplemental resources, such as online videos and written leaflets, to reinforce learning. An earlier study showed that only 40% of patients retain all the information delivered during consultations (39), while a more recent study found that significantly fewer patients felt confident in managing adrenal crisis 6–9 months post-education programme compared to immediately after (*P* < 0.001), indicating a decline in competence and self-assurance over due time (38). This study highlights the importance of adequate knowledge for early symptom recognition of an impending adrenal crisis and a simplified hydrocortisone injection device as crucial factors in supporting patient self-management to prevent adrenal crises. While simplifying the injection device may be challenging to achieve in the short term, annual participation in patient education programmes has been widely recommended for individuals with adrenal insufficiency and their families and caregivers (3, 4, 5, 7, 15).

Many participants in our study encountered delays in the parenteral administration of hydrocortisone when presenting in the emergency setting, despite alerting healthcare staff of their adrenal crisis. Some waited an average 90 min, significantly exceeding the recommended 30-min window for hydrocortisone administration at crisis onset (16). Previous studies indicate that many non-endocrine clinicians lack sufficient knowledge for managing adrenal crises (40, 41, 42, 43), emphasising the need for training on endocrine emergencies to be an integral part of continuous professional development. Strategies such as wider implementation of standardised national and European emergency steroid cards (6, 15, 44) and alert systems in electronic medical records and ambulance protocols (4, 45, 46) can improve early identification and urgent triaging of patients presenting with an adrenal crisis.

### Psychological and emotional impact

Previous adrenal crisis enabled participants to understand their own symptomatology, leading to a prompter action on self-management. In addition, the distress experienced during prior adrenal crises-related hospitalisations prompted many participants to become more proactive in timely self-injection or seeking help to prevent further impending adrenal crises from escalating into full crises. Furthermore, participants’ lived experiences helped them to develop coping strategies to self-manage adrenal crises overtime. This also included other preventative strategies such as carrying an emergency injection kit and steroid card, wear a medic alert bracelet and have extra supply of glucocorticoid tablets. Similar coping and adaptation to living with their condition strategies were reported by patients with primary adrenal insufficiency in an earlier study (35).

Many participants, however, reported intense anxiety when faced with the prospect of self-administering a hydrocortisone injection during an adrenal crisis, even if they had prior training. This was associated with the severely compromised health state during a crisis compounded by the complexity of the injection process, as supported by previous studies (18, 19, 27, 36). Self-confidence may also be hindered by perceived low necessity and concerns about glucocorticoid adverse effects (47, 48), contributing to reluctance in stress dosing or injecting hydrocortisone promptly before the severe symptoms of an adrenal crisis making self-injection impossible.

### Support and dependence on others

This study highlighted that the available tools and strategies to support self-management are inadequate and many individuals living with adrenal insufficiency rely on family members, friends and caregivers to administer hydrocortisone injections or advocate for them during an adrenal crisis. These findings align with previous qualitative research (19, 35). Many parents and caregivers often feel compelled to stay close to their children with adrenal insufficiency, adding logistical, financial and emotional strain to the whole family (19). With around 25% of adrenal crises occurring outside the home or hospital (21), providing caregivers and friends with education and promoting public awareness on the management of adrenal crisis is crucial in enhancing timely access to life-saving hydrocortisone injections. Our participants reported that Patient Advocacy Groups (PAGs) and social media groups were invaluable for learning from others’ experiences and identifying effective strategies for managing adrenal crises. Many valued the resources offered by PAGs, particularly educational resources, peer support and endocrine nurse helpline. A survey of patients with rare endocrine conditions found that 79% relied primarily on PAG resources to enhance their understanding and self-management of their condition (49).

## Strengths and limitations

The qualitative nature of this study allowed for an in-depth analysis of the factors that influence the timely administration of parenteral hydrocortisone to manage adrenal crisis. As this was a qualitative study, findings are not intended to be generalised but may be transferable to similar patient populations or healthcare contexts, and may inform future quantitative studies designed to test and validate these insights in broader populations. However, limitations are acknowledged regarding bias in recruitment with a convenience sampling approach via patient advocacy groups and the diversity of the study population, as all participants were White European females diagnosed with secondary or glucocorticoid-induced adrenal insufficiency. In addition, recruiting participants through patient advocacy groups may have introduced selection bias, as these individuals were likely to be more knowledgeable about their condition and proactive in self-management and adrenal crisis prevention. However, as our findings and previous research suggest, the management of adrenal crises still varies considerably among patients, even when standardised education has been provided, and is often influenced by factors beyond the patient’s control, such as the complexity of the injection process (27). The study was limited to participants who had previously experienced adrenal crises; while this focus was essential to explore specific challenges, including participants without a prior crisis could have provided insights into preventive strategies they may have adopted to avoid adrenal crises. The absence of a standardised definition of adrenal crisis led us to adopt a pragmatic approach, leaving it open to participant interpretation. In addition, the use of virtual interviews required participants to have digital access, which may have excluded some patients.

## Conclusions and implications for practice and research

This study contributes to an in-depth understanding of the lived experiences of people with adrenal insufficiency and the key factors for effectively managing adrenal crises. It highlights the need for a holistic approach involving knowledgeable healthcare professionals, comprehensive patient education and the timely administration of parenteral hydrocortisone in home or community settings.

To improve adrenal crisis management, simpler, more intuitive injection devices, similar to autoinjectors used for anaphylaxis (50), are essential to reduce cognitive and physical demands on patients when self-injecting. However, as this study demonstrated, a simplified hydrocortisone device alone will not fully address these challenges and requires a comprehensive approach involving patients, their families and caregivers, patient advocacy groups and healthcare professionals. Wider implementation of standardised, yet individualised, patient education programmes and self-management strategies is crucial to support crisis prevention, symptom recognition and hydrocortisone administration. These programmes should be grounded in evidence-based practice and behaviour change models, delivered by trained professionals and supported by accessible tools and resources (4, 28, 38, 51). Further research is needed to evaluate the impact of tailored patient education programmes and self-management interventions.

Incorporating adrenal crisis management into clinical education and professional development can help reduce treatment delays and improve care delivery. This should be supported by infrastructure that facilitates prompt recognition of adrenal crisis and response, such as patient alerts in medical records and clear triage protocols for acute care services. Patient advocacy groups play a vital role in delivering effective patient education, supporting self-management and advancing public and policy awareness of adrenal crisis in partnership with healthcare providers, media, community leaders and political stakeholders. Improving awareness among the public and non-medical personnel could also enhance emergency responses, particularly in schools and community settings. Policy changes to authorise trained individuals to administer injections, alongside the development of simplified injection devices and efforts to reduce barriers to hydrocortisone access, can help reduce preventable hospitalisations and fatalities related to adrenal crisis.

## Supplementary materials



## Declaration of interest

The authors declare that there is no conflict of interest that could be perceived as prejudicing the impartiality of the work reported.

## Funding

AC received funding from Barts Charity to complete this study as part of a Masters by Research programme and an Endocrine Research Nurse Award in 2021 from the UK Society for Endocrinology.

## Author contribution statement

AC and SL conceptualised the study and developed the methodology. AC collected and analysed the data, with contributions to data analysis and validation from SL, HY, MC and PO. SL, MC, PO, WD, DT and UD provided supervision and mentorship to AC throughout the research. SL oversaw the study. All authors contributed to manuscript revisions and approved the final version for submission.
