# Supplementary material for: Factors influencing self-management of adrenal crisis in patients with adrenal insufficiency: a qualitative study
Source: Endocr Connect. 2025 Apr 28;14(5):e240651. doi: 10.1530/EC-24-0651 (PMC12060673; doi:10.1530/EC-24-0651)
Supplement: Supplementary file 1 [file supplementary_materials.pdf]

Title: Factors Influencing Self-Management of Adrenal Crises in Patients with Adrenal Insufficiency: A Qualitative Study

Supplementary material: The interview guide

Prior to each interview participants were asked details such as demographics, treatment and condition details, history of hospitalisations and adrenal crisis.

**Q1. Could you tell us about your recent experience with adrenal crisis? How did you feel about it?**	Prompts: What caused/precipitated the adrenal crisis? Where did it happen (i.e., home or travelling)? Did you manage to self-inject the hydrocortisone? Seek for help? What worked and what could have been done better, what barriers they faced, experience of having to give a hydrocortisone injection, response by acute services?
**Q2. How do you find using the emergency steroid injection device?**	Prompts: How do you find the process of self-injecting/administration from preparation to actual self- administration during an adrenal crisis? What worked and what could have been done better?
**Q3. Can you tell us about the training or teaching on sick day rules and emergency hydrocortisone injection techniques you received?**	Prompts: What information resources, what preferences, resources outside specialist services such as GPs, patient charities/support groups, internet. Any concerns or gaps in knowledge? Would more education/support needed to manage future adrenal crisis?
**Q4. Tell me more, overall, what you think would help to manage adrenal crisis better?**	Prompts: injection kit, medic alert/steroid card, what is offered by the clinic, preferred hydrocortisone injection device, calling 999/ambulance, reliance on carers/family or friends. More concerns/gaps in knowledge overall
**Q5. Is there anything else you would like to add/talk about?**	
